# Germline findings in cancer predisposing genes from a small cohort of chordoma patients

**DOI:** 10.1007/s00432-024-05706-5

**Published:** 2024-05-03

**Authors:** Margarita Raygada, Liny John, Anne Liu, Julianne Schultz, B. J. Thomas, Donna Bernstein, Markku Miettinen, Mark Raffeld, Liqiang Xi, Manoj Tyagi, Kenneth Aldape, John Glod, Karlyne M. Reilly, Brigitte C. Widemann, Mary Frances Wedekind

**Affiliations:** 1 Pediatric Oncology Branch, Center for Cancer Research, National Cancer Institute, Bethesda, MD USA; 2 Laboratory of Pathology, Center for Cancer Research, National Cancer Institute, Bethesda, MD USA; 3 NIH Clinical Center (Building 10), 10 Center Drive, Room 1-3750, Bethesda, MD 20892 USA

**Keywords:** Chordoma, Germline, Genetic counseling, Hereditary, Predisposition

## Abstract

**Introduction:**

Chordoma is a rare slow-growing tumor that occurs along the length of the spinal axis and arises from primitive notochordal remnants (Stepanek et al., Am J Med Genet 75:335–336, 1998). Most chordomas are sporadic, but a small percentage of cases are due to hereditary cancer syndromes (HCS) such as tuberous sclerosis 1 and 2 (*TSC1/2*), or constitutional variants in the gene encoding brachyury T (*TBXT*) (Pillay et al., Nat Genet 44:1185–1187, 2012; Yang et al., Nat Genet 41:1176–1178, 2009).

**Purpose:**

The genetic susceptibility of these tumors is not well understood; there are only a small number of studies that have performed germline genetic testing in this population.

**Methods:**

We performed germline genetic in chordoma patients using genomic DNA extracted by blood or saliva.

**Conclusion:**

We report here a chordoma cohort of 24 families with newly found germline genetic mutations in cancer predisposing genes. We discuss implications for genetic counseling, clinical management, and universal germline genetic testing for cancer patients with solid tumors.

## Introduction

Chordomas are rare, slow-growing tumors of skull base and vertebra with an incidence of approximately 300 new cases per year in the United States (McMaster et al. 2001; Wedekind et al. 2021). Most chordomas are sporadic with a few familial brachyury T (Bhadra and Casey 2006) and syndromic (*TSC1/TSC2*) cases reported (Lee-Jones et al. 2004; McMaster et al. 2011). Histologically, chordomas are classified into three subtypes—conventional (most common), de-differentiated (rare), and poorly differentiated (rare) (Fletcher et al. 2013). They are most commonly found in the sacrum/coccyx (50%), followed by skull base (35%), and then mobile spine (15%) (Fletcher et al. 2013).

The molecular characterization of these tumors has been well delineated; nearly all chordomas express T-brachyury (Miettinen et al. 2015), approximately 80% of chordomas have loss of C*DKN2A*, 16% have *PI3K* mutations, 17% have *SWI/SNF* mutations (poorly differentiated and some de-differentiated chordomas), and 10% have *LYST* mutations (Le et al. 2011; Tarpey et al. 2017).

Previous reports examining the association of chordomas with hereditary conditions or germline mutations have focused on the relationship between *TBXT* variants and familial chordoma and have identified an increased risk associated with the presence of *TBXT* variants (Bhadra and Casey 2006; Pillay et al. 2012; Yang et al. 2009). More recent studies have performed germline sequencing in chordoma patients and families to explore new associations with cancer predisposing genes. One such study performed germline whole-exome sequencing in 19 familial chordoma patients and reported defects in *PALB2* and *BRCA2* in 17 patients, the *PALB2* mutation co-segregated with disease in one family (Xia et al. 2022). A second study identified *CHEK2* and *ATM* germline mutations in one patient (Liang et al. 2018). Another study examined germline DNA of 138 chordoma patients of European ancestry and 80 skull-base chordoma patients of Chinese ancestry with close to 23% of cases of both ancestries harboring rare germline variants in relevant signaling pathways such as notochord, mesoderm development, and PI3K/Akt/mTOR (Yepes et al. 2021). Whole exome and genome sequencing was performed in 11 patients with advanced chordomas and found germline mutations in *NBN*, *BRCA2*, and *CHEK2* (Gröschel et al. 2019). More recently, a case report was published of a chordoma patient whose tumor was positive for mutations in the *MSH6* and *MLH1* with subsequent germline testing confirming the presence of the *MLH1* defect with an ultimate diagnosis of Lynch syndrome (Shinojima et al. 2023).

We describe the germline findings of a cohort of 24 patients with chordoma who underwent genetic testing as part of participation in the *M*y *P*ediatric and *A*dult *R*are *T*umor (MyPART) network (https://www.cancer.gov/pediatric-adult-rare-tumor/) *N*atural *H*istory and Biospecimen Acquisition Study for Children and Adults with *R*are *S*olid *T*umors (NHRST) (NCT03739827) at the National Cancer Institute (NCI), Center for Cancer Research (CCR) in response to NCI Cancer Moonshot^SM^ (Singer et al. 2016). MyPART is a team of clinicians and researchers partnering with advocates, patients, and their families to accelerate rare solid tumor research, focusing on patient experience over time.

## Methods

### Patients

All patients were referred by physicians, self-referred, or referred by the Chordoma Foundation (CF). Information about the clinic was available on the NCI Pediatric Oncology Branch and the CF websites. Written informed consent was obtained from all participants or their legal guardians. The NHRST was approved by the NIH Institutional Review Board. Tumor diagnosis was confirmed by pathology review at NCI’s Laboratory of Pathology including immunohistochemical analysis and/or molecular profiling. Imaging studies and standard clinical assessment were performed as part of general participation in the Rare Solid Tumor protocol and the NIH Chordoma Clinics—NCT03739827 (Wedekind et al. 2021).

### Family history

Construction of pedigrees, family data/pedigree information storage, and manipulation were performed using the genetic data management system, Progeny Clinical – Version N from Progeny Genetics (Copyright 2019. Reprinted with permission of Progeny Genetics LLC, Delray Beach, FL, http://www.progenygenetics.com/). By this method, the incidence of primary tumors in 1st, 2nd, and 3rd relatives for each proband was calculated.

### Genetic testing

Germline genetic testing for all participants was performed using genomic DNA extracted from blood or saliva samples. Clinical-grade germline whole-exome sequencing (WES) was performed by GeneDx (GeneDx, Inc) or by the Laboratory of Pathology, National Cancer Institute, National Institutes of Health. Clinical cancer panel gene testing was performed by INVITAE using an 84-gene screening platform on the Invitae Multicancer panel (INVITAE Corporation) which included all cancer-predisposing genes identified by the American College of Medical Genetics and Genomics (ACMG) guidelines. Germline variants tested at the Laboratory of Pathology were from 154 genes with the secondary findings in clinical tumor/normal paired WES. All testing methodologies used next-generation sequencing (NGS) technology; mean depth coverage ranged from 70 to 350X across all genes. The 84 cancer predisposing genes were included in all three methods with equal coverage.

## Results

A total of 24 participants previously diagnosed with chordoma underwent germline genetic testing as described below. There were 18 females and 6 males, ranging in age from 1 to 86 years, with a median age of 16 and mean age of 21 years at enrollment. Twenty participants had conventional chordoma, three poorly differentiated, and one de-differentiated chordoma. Seventeen of the tumors were clival; one located in the extra-axial lumbar spinal canal, three were sacral, one was cranio-cervical, and two were C-Spine.

Table 1 summarizes the results of the germline findings in all 24 participants and their tumor characteristics. A total of 9 out the 24 were positive for heterozygous pathogenic variants identified by either WES or multicancer panel. The patients with positive findings were equally distributed among the three testing methodologies (4 had T/N WES, 5 clinical panel); there were no positive finding in any of the 70 additional genes covered by WES. Three *CHEK2* pathogenic variants (c.1229delC/p.T410fs*;c.1100del/ p.Thr367Metfs*; c.470 T > C/p.Ile157Thr) were identified. All three participants were female (16, 34, and 57 years old) and had conventional chordomas, with two tumors in the clivus and one in the sacrum. One of the patients (57-year-old) was previously diagnosed with NF2 but had no knowledge of her *CHEK2* status. Her family history was not positive for *CHEK2*-related tumors. The second patient (16-year-old) had a negative family history and no other tumors. The father of the third patient (34-year-old) was diagnosed with late onset colon cancer.

**Table 1:** Clinical characteristics and germline variants of participants with chordoma

ID	Gender	Age at diagnosis	Primary chordoma location	Sub-type	Germline pathogenic variant
1	F	32	C/T-Spine	CC	None
2	F	16	Clivus	CC	*CHEK2* c.1229delC/p.T410fs*
3	M	10	Extra-axial/lumbar	CC	*BRCA2* c.4478_4481delAAAG/p.E1493fs*
4	F	5	Clivus/C-Spine	CC	*RET* c.2410G > A p.Val804Met
5	F	34	Clivus	CC	*CHEK2* c.1100del/p.Thr367Metfs*
6	F	29	Clivus	CC	None
7	F	5	Clivus	CC	None
8	F	19	Clivus	PD	None
9	F	5	Clivus	PD	None
10	F	9	Clivus	PD	None
11	M	25	Clivus	CC	None
12	F	57	Sacrum	CC	*CHEK2* c.470 T > C/p.Ile157Thr
13	M	14	Clivus	CC	None
14	F	15	Clivus	CC	*FANCA* c.2852G > A/p.Arg951Gln
15	F	24	Clivus	CC	None
16	F	14	Sacrum	CC	None
17	F	14	Clivus	CC	None
18	M	12	Clivus	DD	*RAD51C* c.706-2A > G/splice site
19	F	31	Clivus	CC	*FH* c.1431_1433dupAAA/p.K477dup
20	M	14	Clivus	CC	None
21	M	25	C-Spine	CC	None
22	F	24	Cranio-cervical	CC	None
23	F	14	Clivus	CC	none
24	F	46	Sacral	CC	*BAP1* c.1975A > T;p.K659Ter

*C/T Spine* cervical-thoracic spine; *C-Spine* cervical spine, *CC* conventional; *PD* poorly differentiated; *DD* de-differentiated

A 5-year-old female with a conventional clival chordoma was positive for a germline pathogenic *RET* variant (c.2410G > A p.Val804Met) and a variant of uncertain significance (VUS) in the *SDHA* gene (c.889C > T p.Pro297Ser). Family history was negative for any *RET*- or *SDHA*-related conditions, both variants were paternally inherited with father (52-year-old) being unaffected.

A 10-year-old male with a conventional lumbar spinal chordoma was found to harbor a pathogenic variant in the *BRCA2* gene (c.4478_4481delAAAG/p.E1493fs*); his family history was non-contributory.

A 15-year-old female with a clival conventional chordoma tested positive for a heterozygous pathogenic mutation in the *FANCA* gene (c.2852G > A/p.Arg951Gln); her family history was significant for a maternal aunt with early onset breast cancer with negative *BRCA1/2*, and maternal grandmother with pancreatic cancer.

A 31-year-old female patient with a clival conventional chordoma was found to have a mutation in the *FH* gene (c.1431_1433dupAAA/p.K477dup). Her mother had late onset colon cancer, and two paternal uncles had melanoma in situ.

A 12-year-old male with a clival de-differentiated chordoma presented with a *RAD51C* pathogenic defect (c.706-2A > G/splice site). This variant was paternally inherited, and his father was unaffected with non-contributory family history.

The last patient to have a significant pathogenic finding was a 46-year-old female with a sacral conventional chordoma and a *BAP1* deleterious mutation (c.1975A > T;p.K659Ter). Her family history was significant for her sister who was diagnosed at 41 years of age with renal cell carcinoma (RCC).

Table 2 summarizes the number of relatives with specific cancer per proband; the family history in all patients was non-contributory including the two patients with paternally inherited germline pathogenic variants (*RET* and *RAD51C*).

**Table 2:** Site-specific family history of cancer by degree of relative

ID	1st Degree (paternal)	2nd Degree (paternal)	3rd Degree (paternal)	1st Degree (maternal)	2nd Degree (maternal)	3rd Degree (maternal)
1						
2					Melanoma (1)	
3		Lymphoma (1) Leukemia (1)				
4		Colon (1)	Breast (1)		Throat (1)	
5	Colon (1)					
6		Colon (1)				
7		Liver (1)	Breast (1)		Melanoma (1)	
8						Unknown Type (2)
9	BCC (1)	Prostate (1)	Prostate (1) Leukemia (1) Liver (1)			
10			Breast (1)			Stomach (1)
11					Colon (1)	
12	Thyroid (1) Lung (2)	Unknown Type (1)				Lymphoma (1)
13		Lung (1)			Lung (1)	Brain Unknown (1)
14			Breast (1) Bladder (1)		Breast (1) Pancreas (1) Skin Unknown (1)	
15		Breast (2)			Breast (1)	Lung (1)
16		Uterine (1)			Liver (1) Colon (1)	
17						
18		Lung (1)		Unknown Type (1)		
19				Colon (1)	Melanoma (2)	Multiple Myeloma (1)
20						
21						
22		SCC Skin (1)		SCC Skin (1)	SCC skin (1) Oligodendroglioma (1)	
23					Melanoma (1) Breast (1)	Lymphoma (1)
24	RCC (1)					

*SCC* squamous cell carcinoma; *BCC* basal cell carcinoma; *RCC* renal cell carcinoma. Parentheses depict number of relatives affected by the specified cancer site

## Discussion

The genetic somatic landscape of chordoma has been well described (Fletcher et al. 2013; Le et al. 2011; Miettinen et al. 2015; Tarpey et al. 2017) but the germline drivers underlying its variability of expression and incomplete penetrance are not fully understood. Here, we report 9 out of 24 patients with non-contributory family history, harboring germline heterozygous deleterious mutation in well-known cancer predisposing genes (*CHEK2, RAD51C, BRCA2, FANCA, RET, FH, and BAP1*). Interestingly, five of these genes (*BAP1*, *FANCA, CHEK2, RAD51C* and *BRCA2*) are part of the BRCA DNA repair pathway (McReynolds et al. 2022). These findings are consistent with previous studies of chordoma patients that have reported germline defects in cancer predisposing genes (*PALB2*, *CHEK2*, *ATM*, *NBN*, *BRCA2* and *MLH*) (Gröschel et al. 2019; Liang et al. 2018; Xia et al. 2022; Yepes et al. 2021)*,* and support their hypothesis that genes involved in DNA repair pathways affecting homologous recombination (HR) may increase susceptibility to chordomas (Gröschel et al. 2019; Shinojima et al. 2023; Xia et al. 2022). *RET* and *FH*, also identified in our cohort, are not directly involved in this pathway. To our knowledge, these two genes have not been associated with increased susceptibility to chordomas.

The family histories (Table 2) in all germline-positive patients were negative for significant findings suggestive of a hereditary cancer syndrome or familial chordoma. This finding is consistent with previous studies that have demonstrated that the vast majority of chordomas are sporadic (Tarpey et al. 2017). All patients except for the *RAD51C* and *FH* heterozygotes, were referred to the NIH without prior knowledge of their germline status and had been followed according to the chordoma screening protocol, as recommended in the published guidelines by the Chordoma Global Consensus Group in 2017 (Stacchiotti et al. 2017). Follow-up surveillance for all cases was adapted post-test according to each defect we identified. The medical history for all 24 patients in this chordoma cohort was otherwise unremarkable.

*CHEK2* mutations were identified in three patients; mutations in this gene are associated with increased risk in breast, colon, and prostate cancer (Hale et al. 2014; Leedom et al. 2016; Shaag et al. 2005; Wang et al. 2015). Risk estimates vary depending on the mutation. One of our patients had the c.470 T > C associated with a lower risk of breast cancer than another patient with the c.1100del (Leedom et al. 2016; NCCN Guidelines® 2022a, b). Two other studies have reported *CHEK2* mutations associated with chordomas (Gröschel et al. 2019; Liang et al. 2018); additional studies are needed to determine causality. Our study is also one of the few to report *BRCA2* mutations in association with chordoma (Gröschel et al. 2019; Xia et al. 2022). Our patient was a 10-year-old male with conventional chordoma and no significant family history of cancer (Table 2, #10). The remaining gene defects in the other five patients are first reported here as germline defects identified in chordoma. All five genes (*BAP1*, *FANCA, RET, FH,* and *RAD51C*) are well-known cancer predisposing genes with incomplete penetrance and variability of expression. All but *FANCA* are penetrant in heterozygotes and their associated conditions are inherited in an autosomal dominant manner.

The *FANCA* gene is involved in an autosomal recessive bone marrow syndrome which causes cancer predisposition in homozygote patients. However, the current data on increased susceptibility to cancer in *FANCA* heterozygote carriers is conflicting and not supportive of a strong effect (McReynolds et al. 2022). More studies are needed to establish risk of cancer for *FANCA* heterozygote carriers.

Pathogenic variants in the *RET* proto-oncogene cause multiple endocrine neoplasia type 2 (MEN2) and familial medullary thyroid cancer (FMTC); penetrance and clinical presentation varies according to the type and location of the variants. Our patient inherited the c.2410G > A p.Val804Met variant from her father who was unaffected. This variant has been described in numerous patients with MEN2 and MTC and is classified as moderate (Eng and Plitt 1999). The clinical manifestations of *RET* variants located in codon 804 are variable even within families (Eng and Plitt 1999), but chordomas have never been reported as part of the phenotype.

Heterozygous mutations in the fumarate hydratase (*FH*) cause *FH* tumor predisposition syndrome, an autosomal dominant condition with increased risk for uterine and cutaneous leiomyomata, renal tumors, and a lower risk of pheochromocytomas and paragangliomas (Kamihara et al. 1993). Homozygosity causes fumarase deficiency, an autosomal recessive condition characterized by failure to thrive, neonatal distress, developmental delay, brain abnormalities, and progressive encephalopathy (Bourgeron et al. 1994). Our patient did not have any symptoms suggestive of *FH* tumor predisposition syndrome, and her family history was negative for chordoma and *FH*-related complications.

*RAD51C* is also involved in DNA repair by initiating HR when DNA damage occurs (Boni et al. 2022) with pathogenic variants conferring higher risk of breast and ovarian cancer (Yang et al. 2020). Our patient’s family history was not significant for familial chordoma, breast, or ovarian cancer.

*BAP1* is a tumor suppressor gene that contains binding domain for *BRCA1* and *BARD1* (Han et al. 2021); germline mutations predispose patients to a variety of cancers including uveal and cutaneous melanoma, mesothelioma, lung adenocarcinoma, meningioma, and renal cell carcinoma (Carbone et al. 2015, 2020). A few patients with missense mutations in *BAP1* have been reported with a neurodevelopmental disorder with variable expression (Kury-Isidor syndrome; KURIS) (Küry et al. 2022) characterized by mild global developmental delay. The patient’s sister suffered from renal cell carcinoma, but no genetic testing was performed; family history was negative for developmental delay.

The genetic etiology of chordoma has not been fully characterized and little is known about factors that increase susceptibility to these tumors. Our study represents an addition to the small pool of published reports of germline testing in chordoma patients that have identified deleterious mutations in cancer predisposing genes (Gröschel et al. 2019; Liang et al. 2018; Shinojima et al. 2023; Xia et al. 2022; Yepes et al. 2021).

The patients described here did not meet criteria for clinical genetic testing; they underwent germline genetic testing as part of a research protocol. Thus, identification of these cancer predisposing variants would have been missed in a clinical setting. Current criteria for germline testing of solid tumor cancer patients are mostly based on positive family history (Esplin et al. 2022; Perkins et al. 2021). However, in recent years, several studies have demonstrated that universal germline genetic testing in unselected cancer patients with solid tumors yields a significant percentage (3.9–56.2%) of positive pathogenic mutations in cancer predisposing genes (Esplin et al. 2022; Jones et al. 2023; Perkins et al. 2021; Samadder et al. 2021; Uson Junior et al. 2022; Uson et al. 2021, 2022). In comparison, our testing on chordoma patients identified pathogenic mutations in 38% of patients, which is within the reported range on other solid tumors. Our findings highlight the potential benefits of universal germline genetic testing in cancer patients with solid tumors.

All chordoma patients in our study received genetic counseling regarding their results, and all 1st degree relatives were offered genetic testing. Preventive measures for these patients would have not been implemented had they not participated in our study. Though it is still too early to establish an association between chordoma and these genetic defects, it is undeniable that the universal germline testing was informative to the patients and their families.

## Conclusions

This study represents another essential contribution to the data supporting the role of these cancer genes in increased susceptibility to chordomas and supports the evidence that universal germline genetic testing for solid tumor cancer patients is beneficial and informative. We examined a cohort of 24 patients with chordoma and found germline mutations in well-known cancer predisposing genes in 38%. The patients all had non-contributory family history and did not meet the criteria for clinical genetic testing. Our findings proved to be significant to the clinical management of this cohort, suggesting potential benefits in the implementation of universal germline testing for solid tumor cancer patients. With future expansion of genetic testing, we aim to better understand the genetic etiology of chordoma and other solid tumors.

## Data Availability

Data are available through controlled access at dbGAP (RRID:SCR_002709) accession phs003143.v1.p1 (http://www.ncbi.nlm.nih.gov/projects/gap/cgi-bin/study.cgi?study_id=phs003143.v1.p1).
